# Can coach emotional support motivate college athletes to train disligently? A moderated mediation model

**DOI:** 10.3389/fpsyg.2026.1790913

**Published:** 2026-04-29

**Authors:** Mengfen Liu, Jiawei Chen, Yang Liu, Bo Zheng, Xinjie Han, Zeng Zhou

**Affiliations:** 1 Department of General Education, Hunan Mechanical and Electrical Polytechnic, Changsha, Hunan, China; 2 School of Physical Education and Arts, Hunan University of Medicine, Huaihua, Hunan, China; 3 Gdansk University of Physical Education and Sport, Gdańsk, Poland; 4 Department of Physical Education, Central South University, Changsha, Hunan, China

**Keywords:** behavioral regulation in exercise, coach emotional support, college athletes, sport confidence, train disligently

## Abstract

**Purpose:**

This study investigates the effects of Coach Emotional Support on diligent training among elite athletes in the Chinese cultural context, as well as the influence mechanism following the introduction of Sport confidence. It also examines the moderating role of Behavioral regulation in exercise in the overall influence mechanism.

**Methods:**

A questionnaire survey was conducted, with 1705 college student-athletes recruited as participants. Furthermore, SPSS 26.0 and AMOS 23.0 were employed to perform correlation analysis, regression analysis, and mediation effect tests on the collected data.

**Results:**

(1) Coach Emotional Support, Sport confidence, and Behavioral regulation in exercise all exerted a significantly positive effect on diligent training behavior among college student-athletes; (2) Sport confidence played a partial mediating role between Coach Emotional Support and the training effort of college student-athletes. The total effect value was 1.023 (*p* < 0.001), and the direct effect value was 0.780 (*p* < 0.001). Within the mediating pathway, the predictive coefficient of Coach Emotional Support for Sport confidence was 0.512 (*p* < 0.001), while that for diligent training was 0.476 (*p* < 0.001); (3) Behavioral regulation in exercise significantly moderated the first stage of the aforementioned mediating pathway, with a moderated mediation index of 0.04 (BootSE = 0.012, LLCI = 0.02, ULCI = 0.067). Conditional indirect effect analysis revealed that as the level of Behavioral regulation in exercise increased, the mediating role of Sport confidence was gradually strengthened.

**Conclusion:**

Coach Emotional Support exerts a significant positive direct effect on diligent training among college student-athletes; Sport confidence acts as a partial mediator between Coach Emotional Support and diligent training in this population; Behavioral regulation in exercise is significantly associated with the strength of the relationships between Coach Emotional Support and Sport Confidence, as well as between Sport Confidence and Train Diligently, reflecting its moderating role in the associative pathways, positively moderating the Coach Emotional Support → Sport confidence pathway and negatively moderating the Sport confidence → diligent training pathway. The present study uncovers the multiple pathways through which Coach Emotional Support influences diligent training among college student-athletes. It provides theoretical foundations and practical implications for enhancing athletes’ diligent training in physical education practice by optimizing emotional support, improving Sport confidence, and strengthening behavioral regulation.

## 1 Introduction

In recent years, global competitive sports have become increasingly intense. The quality of training engagement among college student-athletes directly determines the exertion of their competitive potential and competitiveness in international competitions, thereby driving a steady rise in attention from both academic and sports practice communities toward the key psychological and environmental factors influencing athletes’ diligent training(TD). The Mental Health Toolkit for Elite Athletes, released by the International Olympic Committee (IOC) in 2021, emphasizes that athletes’ mental and physical health are integrated, and a supportive interpersonal environment serves as the core foundation for ensuring athletes maintain long-term high-intensity training. Furthermore, a study on world and Olympic champions found that strong interpersonal relationships and emotional support are key factors for athletes to sustain training continuity and achieve competitive success (Durand-Bush and Salmela, 2002). As a key role that integrates guidance and emotional companionship functions in athletes’ development, coaches’ emotional support behaviors possess unique values that have not yet been fully explored. Thus, it is essential to deeply understand the intrinsic correlation and functional boundaries between Coach Emotional Support(CES) and athletes’ TD.

Existing studies have primarily explored this issue through three lines of inquiry: first, investigating the antecedents of athletes’ TD, focusing on factors such as coach leadership, sports motivation, and team climate (Machida-Kosuga and Kohno, 2023); second, analyzing its consequences for athletes’ competitive performance, psychological resilience, and career sustainability (Trigueros et al., 2019; Barlow and Banks, 2014); third, examining the mediating mechanisms or contextual moderators between antecedent and outcome variables (Zhang and Fan, 2025; Li et al., 2025; Garrido-Muñoz et al., 2024). However, existing research has often conflated CES with instrumental support (e.g., technical guidance and information feedback), failing to clarify its unique connotations of emotional catharsis, belief enhancement, and relational empathy, nor to precisely define the core dimensions of this construct. Most studies have only tested the direct relationship between coach support and training engagement, without systematically verifying the mediating role of SC between them, and have overlooked the interactive effect of Behavioral regulation in exercise (BRE) on the mediating pathway. This makes it difficult to explain the intrinsic logic by which CES is converted into training motivation. These limitations have hindered our ability to address the key question of whether and how CES can effectively promote TD among college student-athletes, as well as its functional boundaries and moderating conditions. Thus, the rationale and objective of this study are to take coaches’ emotional support as the entry point, analyze the path relationships between factors influencing college student-athletes’ TD, and further propose behavioral strategies to promote their TD.

### 1.1 The relationship between coach emotional support and train disligently

CES refers to the psychological support and empowerment provided by coaches to athletes through emotional care, belief encouragement, empathetic understanding, and other means in training and daily life (Simons and Bird, 2023; Ferreira et al., 2024). According to Self-Determination Theory (SDT), when an individual’s three basic psychological needs—autonomy, competence, and relatedness—are satisfied, intrinsic motivation is aroused, thereby promoting goal-directed positive behaviors (Ryan and Deci, 2000). The multi-dimensional nature of CES enables it to precisely match and satisfy these three needs: First, the coach’s empathetic understanding directly meets the athlete’s autonomy needs (Lorimer and Jowett, 2013). As the central authoritative figure in the training context, the coach respects the athlete’s training opinions, supports the independent adjustment of training plans, and avoids autocratic decision-making, thereby allowing athletes to feel autonomy and voice in their training participation. For college athletes who are in the stage of pursuing independence, this empathetic interaction breaks the traditional top-down coaching model, allowing them to perceive training behaviors as autonomous choices rather than passive acceptance, thus enhancing their intrinsic motivation to participate in training. Second, the coach’s belief encouragement effectively satisfies the athlete’s competence needs (Mageau and Vallerand, 2003). The coach’s affirmation of training progress, recognition of potential, and encouragement in the face of challenges help athletes develop a positive perception of their athletic abilities. During high-intensity training and dual-task conflicts, athletes are prone to doubting their own abilities. Belief encouragement plays a positive feedback role, reinforcing a growth mindset that athletic ability can be enhanced through training, enabling athletes to continually accumulate a sense of achievement in training, thus consolidating their confidence in completing training tasks. Third, the coach’s emotional care strongly satisfies the athlete’s relatedness needs (Lafrenière et al., 2011). Ongoing emotional care, listening to psychological pressure, and empathizing with difficulties help establish a strong emotional bond between the coach and the athlete (Rocchi and Pelletier, 2018; Liu et al., 2025). Moreover, these three traits do not operate independently; instead, they synergistically enhance the satisfaction of basic psychological needs. For example, empathetic understanding lays the foundation for emotional trust, making athletes more receptive to belief encouragement; emotional care strengthens the emotional bond, making the autonomy support in belief encouragement more strongly perceived. This multidimensional and synergistic satisfaction mechanism ensures that CES comprehensively activates the athlete’s intrinsic motivation, thereby promoting TD behavior. Existing studies have found that perceived CES can significantly and positively predict athletes’ training engagement (López-Walle et al., 2012); in addition, coaches’ emotional care can indirectly promote their training persistence by enhancing athletes’ intrinsic motivation (Teixeira et al., 2012). For college student-athletes, their training behaviors are not entirely driven by professional contracts but rely more on intrinsic motivation and psychological identification. The positive psychological environment constructed by CES can effectively weaken the negative impact of academic-training conflict, strengthen the cognitive link of “TD - ability improvement - self-actualization”, and thereby promote their willingness to exert effort in training. In summary, Hypothesis 1 is proposed in this study: CES is positively correlated with TD among college student-athletes.

### 1.2 The mediating role of sport confidence

SC is defined as a stable, positive cognitive belief and psychological state held by athletes in competitive training and competition contexts, reflecting their perceptions of their own athletic abilities, potential to cope with challenges, and likelihood of achieving training goals. It constitutes a core psychological capital that modulates athletes’ training behaviors and competitive performance (Vealey, 1986; Hays et al., 2009). As a key psychological bridge linking environmental support to behavioral performance, the formation and development of SC are significantly shaped by external supportive environments and directly modulate an individual’s training motivation and engagement levels. This logical framework is highly consistent with the core tenets of Self-Determination Theory (SDT). First, CES serves as a key antecedent for enhancing SC among college student-athletes. Based on Self-Determination Theory, coaches’ emotional support provides a core psychological foundation for the development of SC by fulfilling athletes’ needs for competence, relatedness, and autonomy. Coaches’ emotional care, recognition of athletes’ values, and empathetic understanding can effectively mitigate athletes’ fear of failure and strengthen their growth mindset that athletic abilities can be enhanced through training (López-Walle et al., 2012). Previous studies have confirmed that coaches’ emotional support behaviors can significantly and positively predict athletes’ SC; particularly for college student-athletes facing dual-role conflicts, the secure psychological environment fostered by such emotional support serves as a core safeguard for maintaining their positive perceptions of their own athletic abilities (Wylleman, 2000; Contreira et al., 2019). As a core psychological capital, SC directly modulates an individual’s motivational intensity and frustration tolerance. Athletes with high SC are more inclined to frame training challenges as opportunities for ability enhancement rather than threatening events, thereby demonstrating greater initiative and persistence in training (Carpentier and Mageau, 2016). For college student-athletes, their training behaviors are driven more by intrinsic motivation, and SC can further strengthen this intrinsic motivation, enabling them to maintain a high level of training engagement even when confronted with various adversities. A growing body of empirical research has further confirmed that SC is a key predictor of athletes’ training engagement and persistence; athletes with high confidence are more likely to invest extra effort in high-intensity training and better tolerate the physical and mental demands imposed by training (Machida et al., 2012; Lorimer and Jowett, 2013). In summary, Hypothesis 2 is proposed in this study: SC plays a mediating role between CES and TD among college student-athletes.

### 1.3 The regulatory role of behavioral regulation in exercise

The mediating pathway through which CES influences TD among college student-athletes via SC may be moderated by other factors. BRE is defined as an individual’s psychological process and capacity to actively plan, monitor, and dynamically adjust their training goals, implementation strategies, emotional responses, and behavioral performance in competitive training contexts. Its core manifestation lies in the autonomous regulatory tendency to flexibly optimize behavioral patterns and address training dilemmas based on training needs (Wilson et al., 2012; Markland and Tobin, 2004). This construct is inherently consistent with the multi-dimensional measurement logic of the REQ-2 scale: the five motivational regulation styles (from AM to IM) reflect the gradual development of regulatory autonomy—individuals with higher total scores tend to adopt more self-determined motivational regulation (e.g., ID and IM), and thus exhibit stronger capabilities in active planning, dynamic monitoring, and strategy adjustment during training. In contrast, individuals with lower total scores are more likely to be driven by non-self-determined motivation (e.g., ER and AM), leading to insufficient autonomous regulation capabilities. Therefore, using the total score of REQ-2 to assess BRE can effectively capture the core connotation of the construct, realizing the alignment between measurement operation and theoretical definition. BRE can significantly predict the formation and stability of SC among college student-athletes. Existing studies have found that BRE is significantly positively correlated with perceived coach support and SC (Edmunds et al., 2006; Contreira et al., 2019). Individuals with high levels of BRE possess more robust resource integration capabilities and self-regulation strategies, thereby enhancing their sensitivity to identifying CES and improving the efficiency of converting supportive information into self-ability recognition; in the process by which CES acts on SC, compared with individuals with low levels of BRE, those with high levels exhibit a stronger willingness to actively capture signals of CES and are more capable of internalizing external emotional support into the positive cognition that “coaches recognize my abilities”, thereby mitigating the negative impacts of self-doubt during training. In contrast, low levels of BRE act as maintaining factors for the deficiency and fluctuation of SC. Low levels of BRE can be regarded as the inaccurate processing of external emotional support information by individuals. Those characterized by low BRE exhibit insufficient autonomous regulatory and information interpretation capabilities, failing to correctly identify the risks of neglecting CES and failing to convert it into self-affirmation, which leads to a lack of awareness and motivation to utilize external support to enhance SC. In summary, Hypothesis 3 is proposed: BRE may play a moderating role in the pathway through which CES influences TD among college student-athletes.

Athletes’ SC influences the sustainability and depth of engagement in their TD, and this effect may be moderated by BRE. Individuals with low levels of BRE are more prone to experiencing ambiguity regarding training goals and helplessness in coping with setbacks, thereby generating more discrepancies in cognition-behavior translation—that is, although they possess a certain level of SC, they struggle to translate their intrinsic belief that they can achieve training goals into actual training engagement. According to Self-Determination Theory (SDT), this translation discrepancy perpetuates a negative feedback loop: failure to translate SC into action → suboptimal training outcomes → diminished confidence → further withdrawal. Ultimately, this leads college student-athletes to adopt behaviors such as training slackness, passive compliance, and even training avoidance to terminate the persistent frustration and self-efficacy depletion (Liu et al., 2025). In contrast, individuals with high levels of BRE excel at enhancing the efficiency of translating SC into training behaviors by proactively decomposing training goals, dynamically adjusting implementation strategies, and timely regulating negative emotions. This capability enables them to replace passive withdrawal with rational approaches such as phased breakthroughs and targeted improvements, thereby maintaining the sustainability and depth of training engagement and converting SC into sustained motivation for overcoming difficulties. In summary, Hypothesis 4 is proposed in this study: BRE may play a moderating role in the pathway through which SC influences TD among college student-athletes.

**Figure 1:**
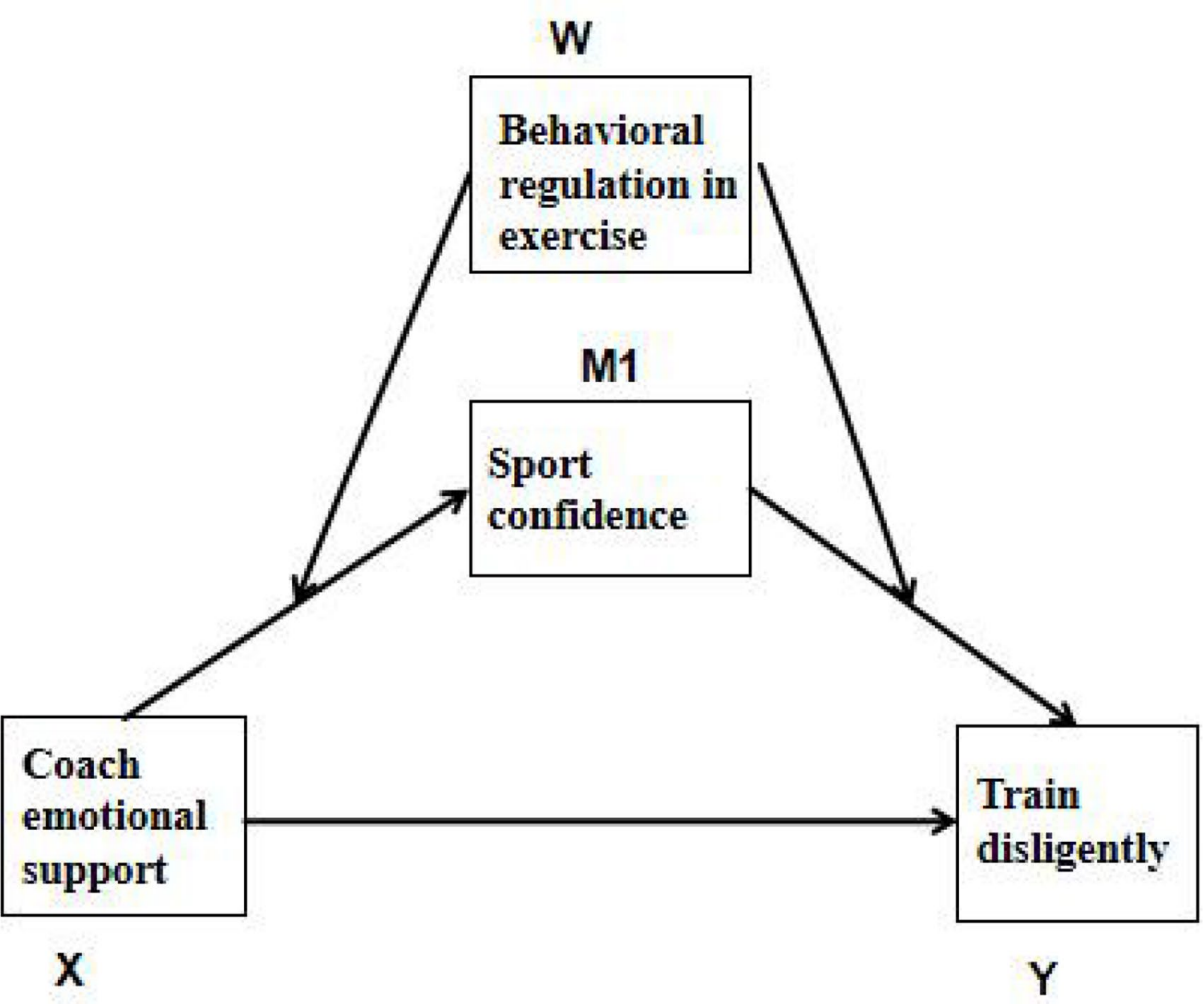
Diagram of the theoretical model.

In summary, a moderated mediation model is proposed in this study, which aims to investigate the effect of CES on TD among college student-athletes, as well as the mediating role of SC and the moderating role of BRE (see Figure 1). The model is intended to provide theoretical foundations and practical implications for promoting TD among college student-athletes.

## 2 Methods

### 2.1 Participants and procedures

From September to December 2025, a stratified cluster sampling method was employed, and online questionnaires were distributed and collected via the Wenjuanxing platform. The study sample comprised student-athletes from six sports universities across Hunan, Guangxi, and Henan provinces, covering major sports such as ball games, athletics, martial arts, and other key disciplines. Participants were aged between 17 and 22 years, with 991 males (58.12%) and 714 females (41.88%). The research protocol was approved by the Ethics Committee of Hunan Mechanical & Electrical Polytechnic (Approval No. 2025121), adhering strictly to the ethical guidelines of the Declaration of Helsinki. Informed consent procedures were implemented stratified by age to ensure compliance with ethical requirements for different age groups. For participants aged 18–22 years old (adults): Electronic written informed consent was obtained directly from the participants themselves. The consent form clearly outlined the research purpose, data collection methods, anonymization measures, right to withdraw at any time without prejudice, and data usage scope. Participants confirmed their consent by clicking the “Agree” button before initiating the questionnaire. For participants aged 17 years old (minors): A two-step consent procedure was adopted. First, electronic written informed consent was obtained from the participants’ parents or legal guardians via the class teachers, who provided detailed explanations of the research protocol and ethical guarantees. Second, after obtaining parental/guardian consent, the participants themselves provided electronic written assent (acknowledging their understanding of the research and voluntary participation). Only after both parental/guardian consent and participant assent were confirmed did the minors gain access to the questionnaire. The research team provided standardized training for the class teachers responsible for implementation, clarifying instructions and key considerations for age-stratified consent collection. The survey design took approximately 15 min, incorporating three attention-check questions to eliminate invalid responses. After the class teachers collected the surveys online, two independent researchers exported and cross-checked the data. A total of 1823 surveys were collected, and after eliminating invalid and incomplete responses through attention-check questions and logical validation, 1705 valid samples were retained, resulting in a response rate of 93.52%.

### 2.2 Measuring tools

#### 2.2.1 Coach emotional support

The CES Scale was adapted from the questionnaire developed by Wei and Xu (2022), with the term “teacher” in the original scale replaced by “coach.” The original scale was designed for the occupational context, and the study revised the core term “teacher” to “coach” and conducted linguistic and cultural adaptation for the sports context, with the steps consistent with the CES Scale: forward translation → back translation → expert panel review → pilot testing. The original scale was translated into Chinese and culturally and linguistically adapted to the Chinese context, with the semantic equivalence of all items maintained throughout the process. The final Chinese version of the scale was consistent with the original items and scoring protocol. The scale comprises 7 items (e.g., “My coach cares deeply about us”), with each item rated on a 5-point Likert scale ranging from 1 (strongly disagree) to 5 (strongly agree). The total score of the scale is calculated as the sum of the scores of 7 items, higher scores indicate a greater perceived level of coach emotional support. In the present study, the CES Scale yielded a Cronbach’s *α* coefficient of 0.889, demonstrating excellent internal consistency reliability.

#### 2.2.2 Sport confidence

The Sport Confidence Scale, developed by Shen (2004) was adopted in this study. It comprises 8 items and consists of two dimensions: items 1, 3, 5, and 7 belong to the athletic task confidence dimension, while items 2, 4, 6, and 8 pertain to the athletic coping confidence dimension. A 1–6 rating scale was used for scoring, with the total score calculated as the sum of all 8 items. Higher total scores indicate stronger levels of SC. In the present study, the Cronbach’s *α* coefficient for the athletic task confidence dimension was 0.644; that for the athletic coping confidence dimension was 0.642; and the overall Cronbach’s α coefficient of the scale reached 0.789, demonstrating good internal consistency reliability.

#### 2.2.3 Behavioral regulation in exercise

The Regulation in Exercise Questionnaire-2 (REQ-2), validated by Liu et al. (2015), was adopted in this study. It consists of 18 items, with each item rated on a 5-point Likert scale ranging from 0 (completely inconsistent) to 4 (completely consistent). This scale assesses five regulatory styles of exercise behavior: Amotivation (e.g., “I think exercising is a waste of time”), External Regulation (e.g., “I exercise because other people say I should”), Introjected Regulation (e.g., “I feel guilty when I don’t exercise”), Identified Regulation (e.g., “it’s important to me to exercise regularly”), and Intrinsic Motivation (e.g., “I find exercise a pleasurable activity”). The total score of BRE is the sum of all 18 items, with higher scores indicating a more adaptive and integrated level of exercise behavioral regulation. In the present study, the Cronbach’s *α* coefficient for Amotivation was 0.937; that for External Regulation was 0.941; that for Introjected Regulation was 0.923; that for Identified Regulation was 0.623; that for Intrinsic Motivation was 0.871; and the overall Cronbach’s α coefficient of the scale reached 0.930, demonstrating excellent internal consistency reliability.

#### 2.2.4 Train disligently

The TD Scale was adapted from the instrument developed by De Cooman et al. (2009), with the term “work” in the original scale replaced by “training.” The original scale was designed for the occupational context, and the study revised the core term “work” to “training” and conducted linguistic and cultural adaptation for the sports context, with the steps consistent with the Coach Emotional Support Scale: forward translation → back translation → expert panel review → pilot testing. The original scale was translated into Chinese and culturally and linguistically adapted to the Chinese context, with semantic equivalence of all items maintained throughout the process. The final Chinese version of the scale was consistent with the original items and scoring protocol. This scale comprises 10 items. Sample items include “I devote substantial effort to the tasks I engage in” and “I do my utmost to complete the tasks that I am required to perform”. Participants responded to each item using a 6-point Likert scale, ranging from 1 (strongly disagree) to 6 (strongly agree). The total score of the scale is calculated as the sum of the scores of 10 items, higher total scores indicate greater levels of effort exerted by individuals during training. In the present study, the TD Scale yielded a Cronbach’s *α* coefficient of 0.914, demonstrating excellent internal consistency reliability.

### 2.3 Statistical analysis

Descriptive statistics and correlation analysis were conducted using SPSS version 26.0, while the PROCESS macro was employed to test for mediation effects, and AMOS 23.0 was used for confirmatory factor analysis (CFA) to verify the validity of the measurement model. Given the different response formats of the four scales (Likert 1–5, 1–6, 0–4), all scale scores were standardized by Z-score before statistical analysis to eliminate the influence of dimensional differences and ensure the comparability of the analysis results. To further verify the robustness of the moderating effect and respond to the methodological suggestion of using self-determination indices, we additionally calculated the Self-Determination Index (SDI) for the REQ-2 scale, following the weighted scoring method recommended by Ryan and Deci (2024) and Markland and Tobin (2004): SDI = (IM × 3) + (ID×2) + (IR × 1) + (ER × 0) + (AM×(−1)). Higher SDI scores indicate higher levels of self-determined motivation. We conducted parallel moderated mediation analyses using both the total score and SDI of BRE, with results reported in Section 3.3. The results of the Harman’s single-factor test revealed that 13 factors with eigenvalues greater than 1 were extracted. The first factor accounted for 35.963% of the total variance, which is below the 40.00% threshold, indicating that common method bias was not a significant concern in this study (Tang and Wen, 2020).

#### 2.3.1 Data preprocessing

Variable coding rules: All scales used in this study have no reverse-coded items; all items are positively coded, meaning the higher the item score, the higher the corresponding construct level. The five dimensions of BRE are retained according to the original scoring rules of the scale, with the total score being the sum of the item scores for each dimension. A higher total score indicates a stronger BRE ability.

Mean centering: To optimize the stability of the moderation effect analysis and avoid multicollinearity interference, the continuous variables CES and BRE were mean-centered. After centering, the variables have a mean of 0, and the standard deviation remains consistent with the original variables. The interaction terms were calculated based on the centered variables. Upon inspection, the variance inflation factors (VIF) of all centered variables ranged from 1.2 to 2.8, which is well below the critical value of 10.0, indicating that multicollinearity issues can be ignored.

Definition of coefficient types: In this study, the regression analysis tables report standardized β coefficients, while the mediation effect path diagram reports unstandardized coefficients. The conditional indirect effects and moderated mediation indices report unstandardized effect values (Effect), along with SE and CI.

### 2.4 Measurement model validity test

To verify the structural validity, convergent validity, and discriminant validity of the measurement scales (considering semantic adaptations and mixed response formats), confirmatory factor analysis (CFA) was conducted using AMOS 23.0 with maximum likelihood estimation. Composite reliability (CR), average variance extracted (AVE), and heterotrait-monotrait ratio (HTMT) were calculated to comprehensively evaluate validity. The results are shown in Tables 1, 2.

**Table 1:** CFA fit indices and convergent validity of measurement model.

Constructs	Items	Factor loadings (*λ*)	CR	AVE
Coach emotional support	7	0.621~0.894	0.892	0.568
Sport confidence	8	0.583~0.812	0.791	0.425
Behavioral regulation in exercise	18	0.657~0.946	0.935	0.592
Train diligently	10	0.682~0.903	0.916	0.547
Model fit indices	—	—	χ2/df = 3.217	CFI = 0.928
—	—	—	TLI = 0.919	RMSEA = 0.048 (90%CI: 0.045~0.051)
—	—	—	SRMR = 0.052	—

**Table 2:** Discriminant validity (HTMT values).

Constructs	CES	SC	BRE	TD
Coach emotional support (CES)	—	0.512	0.703	0.765
Sport confidence (SC)	—	—	0.456	0.689
Behavioral regulation in exercise (BRE)	—	—	—	0.621
Train diligently (TD)	—	—	—	—

Structural Validity: The four-factor measurement model exhibited excellent fit (χ^2^/df = 3.217 < 5; CFI = 0.928 > 0.90; TLI = 0.919 > 0.90; RMSEA = 0.048 < 0.06; SRMR = 0.052 < 0.08), meeting the recommended standards for structural equation modeling. This confirms that the scales maintain clear construct differentiation despite semantic adaptations and mixed response formats.

Convergent Validity: All constructs had CR values ranging from 0.791 to 0.935 (> 0.70), and AVE values ranging from 0.425 to 0.592 (> 0.40). Notably, BRE (a mature scale) achieved CR = 0.935 and AVE = 0.592, significantly exceeding critical thresholds, verifying the excellent convergent validity of adapted scales in the Chinese college student-athlete sample.

Discriminant Validity: All HTMT values were < 0.85, indicating no severe construct overlap. This confirms that each scale measures a distinct theoretical construct, despite semantic adaptations to the sports context.

## 3 Research results

### 3.1 Descriptive statistics and correlation analysis

The means, standard deviations, and correlation coefficients of all variables are presented in Table 3. Descriptive statistics indicate that all variables exhibit reasonable value distributions with no evidence of extreme outliers, thereby providing a sound data foundation for subsequent hypothesis testing. Correlation analysis results reveal that significant positive correlations exist among all core variables (*p* < 0.01), with detailed findings summarized as follows: (1) A significant positive correlation was observed between CES and SC (r = 0.469, *p* < 0.01), indicating that higher levels of perceived CES among college student-athletes are associated with stronger SC; (2) A significant positive correlation was identified between CES and BRE (r = 0.671, *p* < 0.01), suggesting that high levels of CES may facilitate the development of more effective BRE capabilities among athletes; (3) A strong significant positive correlation was detected between CES and TD (r = 0.737, *p* < 0.01), which preliminarily verifies the positive impact of CES on training engagement among college student-athletes; (4) A significant positive correlation was found between SC and TD (r = 0.637, *p* < 0.01), demonstrating that higher levels of SC among athletes correspond to greater levels of TD engagement; (5) A significant positive correlation was established between BRE and TD (r = 0.583, *p* < 0.01), indicating that effective BRE may positively predict the level of training engagement among athletes; (6) A significant positive correlation was also confirmed between SC and BRE (r = 0.403, *p* < 0.01), The positive correlations among the core variables provide the foundation for subsequent mediation and moderation effect tests. It should be noted that in the subsequent regression analysis, the signs of the standardized β coefficients for certain variables appear contradictory to the correlation coefficients; however, this discrepancy arises from the mean-centering of continuous variables (as explained in Section 3.3). In essence, this still reflects the positive relationship between the variables, and the data logic remains fully consistent.

**Table 3:** Table of descriptive statistics and correlation analysis.

	M	SD	Coach emotional support	Sport confidence	Behavioral regulation in exercise	Train disligently
Coach emotional support	22.586	5.285	1			
Sport confidence	26.358	5.769	0.469**	1		
Behavioral regulation in exercise	49.762	9.309	0.671**	0.403**	1	
Train disligently	34.052	7.337	0.737**	0.637**	0.583**	1

*p < 0.05, **p < 0.01.

### 3.2 Mediating effect analysis

**Figure 2:**
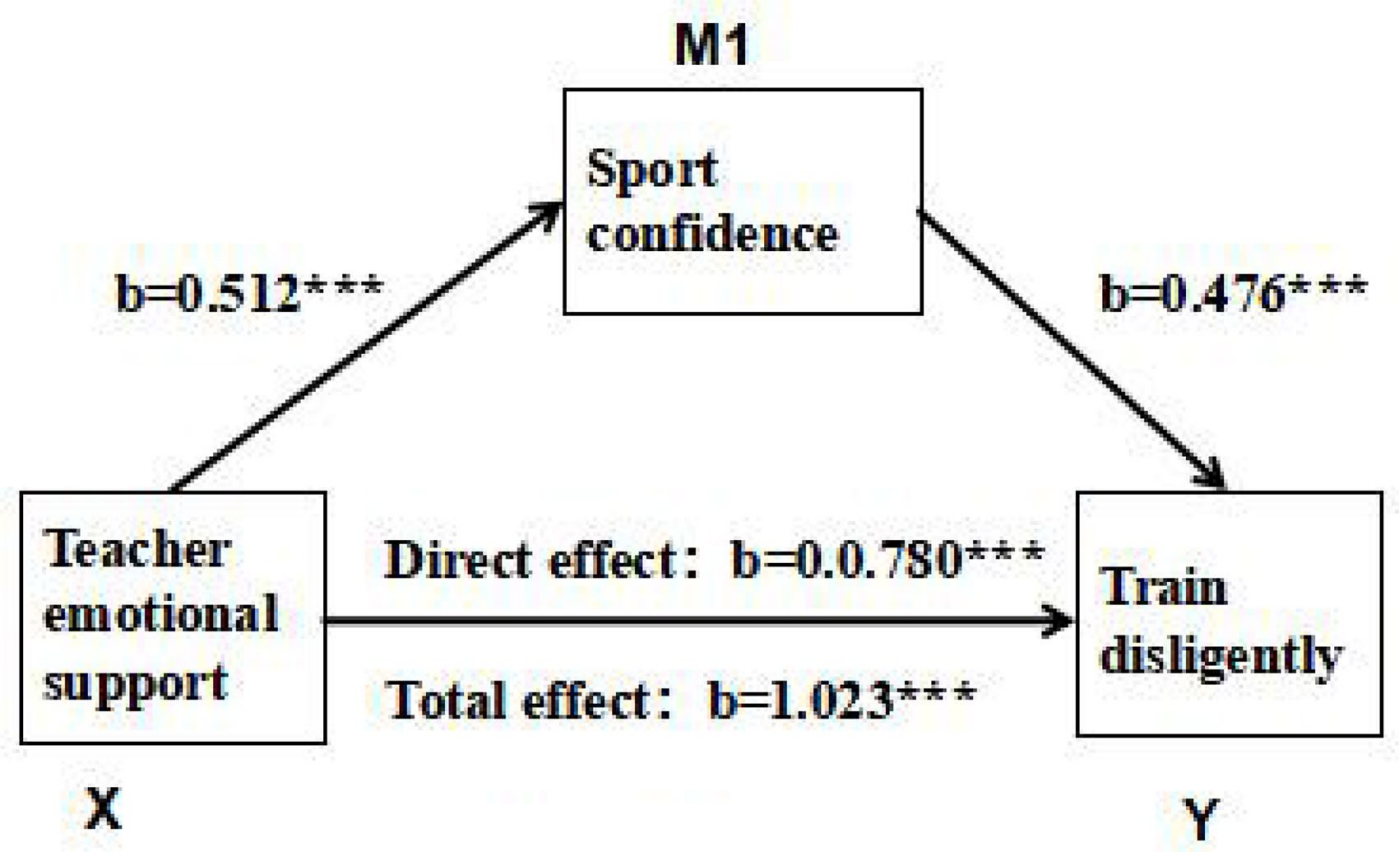
Paths of the mediating effect.

To examine the mediating role of SC between CES and TD among college student-athletes, a mediation effect test was conducted using hierarchical regression analysis combined with the Bootstrap resampling procedure (5,000 resamples), with results presented in Figure 2. Results of the mediation effect test revealed that the total effect of CES on TD among college student-athletes was significant (b = 1.023, *p* < 0.001), indicating that CES can directly and positively predict the level of TD in this population. Further mediating pathway analysis demonstrated that this effect was exerted through two pathways: first, the direct effect pathway: after incorporating SC as the mediating variable, the direct predictive effect of CES on TD remained significant (b = 0.780, *p* < 0.001), indicating that CES is significantly and positively associated with TD among college student-athletes, and the cross-sectional data shows a stable positive predictive relationship; second, the mediating effect pathway: CES significantly and positively predicted SC (b = 0.512, *p* < 0.001), and SC further significantly and positively predicted TD (b = 0.476, *p* < 0.001). Further, the mediating effect was calculated: CES exerts a mediating effect on TD via SC, computed as 0.512 (CES → SC) × 0.476 (SC → TD) ≈ 0.243. The proportion of the mediating effect relative to the total effect is 0.243/1.023 ≈ 23.75%. The total effect (1.023) is thus the sum of the direct effect (0.780) and the mediating effect (0.243). The logical consistency of these values supports the validity of the partial mediating effect. These results indicate that SC plays a partial mediating role between CES and TD among college student-athletes; that is, CES can not only directly facilitate TD among college student-athletes but also indirectly promote training engagement by enhancing their SC. These findings are fully consistent with the theoretical expectations of Hypothesis 2.

### 3.3 Moderated mediation effect test

A moderated mediation effect test was performed using PROCESS Macro Model 58 proposed by Hayes (2013). The Bootstrap resampling method was employed with 5,000 repetitions to estimate confidence intervals, and the results are presented in Tables 4–7.

**Table 4:** Regression analysis of variable relationships.

	Train disligently		Sport confidence	
	*β*	*t*	*β*	*t*
Coach emotional support	0.74	26.79***	−0.895	−9.175***
Behavioral regulation in exercise	0.459	10.415***	−0.492	−10.723***
Coach emotional support*behavioral regulation in exercise			0.025	13.864***
Sport confidence	1.129	14.889***		
Sport confidence*behavioral regulation in exercise	−0.014	−9.138***		
R^2^	0.674		0.312	
F	878.488***		256.982***	

***p < 0.001; All coefficients in the table are standardized regression coefficients. Continuous predictor variables were mean-centered, and interaction terms were constructed based on the mean-centered variables. All scales are free from reverse-coded items, and variable encoding is entirely forward.

**Table 5:** Direct effect.

Effect	SE	***t***	LLCI	ULCI
0.74	0.028	26.79	0.685	0.794

**Table 6:** Conditional indirect effect.

Mediator		Level value	Effect	LLCI	ULCI
Sport confidence	˗1SD	40.452	0.074	0.009	0.125
	M	49.762	0.164	0.124	0.206
	+1SD	59.071	0.195	0.14	0.246

**Table 7:** The moderated mediation index.

Regulating variable	Mediating variable	Index	BootSE	LLCI	ULCI
Behavioral regulation in exercise	Sport confidence	0.04	0.012	0.02	0.067

Table 4 presents the results of multiple regression analyses predicting the mediating variable (SC) and the dependent variable (TD). Both regression models passed the significance test and exhibited good model fit. In the regression model predicting the mediating variable (Sport confidence, SC): the model exhibited significant overall explanatory power (R^2^ = 0.312, *F* = 256.982***). It is critical to note that Coach Emotional Support (CES) and Behavioral regulation in exercise (BRE) were mean-centered prior to analysis, and all reported coefficients are standardized β. The main effect of CES was significant (*β* = −0.895, t = −9.175***), which reflects the positive predictive role of CES on SC: when CES scores are below the sample mean, SC levels decrease significantly; conversely, when CES scores are above the sample mean, SC levels increase significantly. This interpretation is fully consistent with the zero-order correlation coefficient between CES and SC (r = 0.469**, Table 2), confirming the intrinsic positive relationship between the two variables. Similarly, the main effect of BRE was significant (*β* = −0.492, t = −10.723***), which also reflects the positive predictive role of BRE on SC (consistent with r = 0.403**, Table 2). The core interaction term “CES × BRE” exerted a significantly positive predictive effect on SC (*β* = 0.025, t = 13.864***), indicating that BRE significantly positively moderates the relationship between CES and SC—i.e., the higher the BRE level, the stronger the positive predictive effect of CES on SC. Hypothesis 3 was empirically supported. In the regression model predicting the dependent variable (TD): the model demonstrated excellent overall explanatory power (R^2^ = 0.674, *F* = 878.488***, *p* < 0.001). The main effect of CES was significantly positive (*β* = 0.74, t = 26.79***, *p* < 0.001), the main effect of BRE was significantly positive (*β* = 0.459, t = 10.415***, *p* < 0.001), and the main effect of SC was significantly positive (*β* = 1.129, t = 14.889***, *p* < 0.001). The key interaction term “SC × BRE” exerted a significantly negative predictive effect on TD (*β* = −0.014, t = −9.138***, *p* < 0.001), indicating that BRE significantly moderates the relationship between SC and TD. Hypothesis 4 was empirically supported.

Table 5 reports the results of the direct effect of CES on TD among college student-athletes. The data demonstrate that after controlling for the mediating variable (SC), the moderating variable (BRE), and their interaction terms, the direct effect size of CES on TD was 0.74 (SE = 0.028, t = 26.79). The 95% confidence interval [LLCI = 0.685, ULCI = 0.794] excluded zero, indicating that CES exerts an independent and significant positive predictive effect on TD among college student-athletes, which further corroborates the core conclusion of Hypothesis 1.

To clarify the specific differences in indirect effects across varying levels of BRE, Table 6 presents a conditional indirect effect analysis stratified into low, medium, and high levels, based on the mean of BRE plus or minus one standard deviation (-1SD, M, +1SD). The results indicate that the conditional indirect effects were significant across all three moderating levels (95% confidence intervals excluded zero in all instances), with effect sizes exhibiting a graded increasing trend. The observed increasing trend of the conditional indirect effect is consistent with the positive moderating logic of BRE. The larger increase observed from the low to medium level may be attributable to a threshold effect in the regulatory capacity of the low-BRE group, such that when BRE levels increase from low to medium, the efficiency with which athletes translate coaches’ emotional support is significantly enhanced; whereas further increases from medium to high levels result in efficiency approaching saturation, with increments becoming gradually attenuated. This pattern aligns with the law of diminishing marginal returns in self-regulatory capacity in sports psychology (Hagger and Chatzisarantis, 2008), further validating the authenticity of the moderating effect.

Table 7 reports the results of the moderated mediation index test based on Model 58. The moderated mediation index of BRE on the “X → M → Y” pathway was 0.04 (BootSE = 0.012), and the 95% confidence interval [LLCI = 0.020, ULCI = 0.067] excluded zero. This result indicates that the moderated mediation model constructed in this study was statistically significant overall. Combined with the results of interaction effects and conditional indirect effects reported earlier, it can be concluded that BRE ultimately synergistically enhanced the magnitude of the indirect effect through which CES influences TD via SC, by positively moderating the first stage and negatively moderating the second stage of the mediating pathway. This finding fully supports the core theoretical framework of the study, namely that BRE serves as a key boundary condition for the “X → M → Y” pathway.

To confirm the robustness of the moderating effect, parallel analyses were conducted using both the total score of BRE and the SDI index. The results showed high consistency between the two scoring methods: When using SDI as the moderating variable, the interaction term “CES × SDI” still exerted a significantly positive predictive effect on SC (*β* = 0.028, t = 14.217***, *p* < 0.001), consistent with the result of the total score (*β* = 0.025, t = 13.864***, *p* < 0.001); The interaction term “SC × SDI” exerted a significantly negative predictive effect on TD (*β* = −0.016, t = −9.572***, *p* < 0.001), consistent with the result of the total score (*β* = −0.014, t = −9.138***, *p* < 0.001); The moderated mediation index based on SDI was 0.043 (BootSE = 0.013, 95%CI = [0.021, 0.070]), which was consistent with the index based on the total score (0.04, BootSE = 0.012, 95%CI = [0.020, 0.067])—both excluded zero, confirming the validity of the moderated mediation model. The consistency of the results between the two scoring methods fully verifies the robustness of the moderating effect of BRE. Since the total score is the mainstream scoring method of the REQ-2 scale, is more consistent with the theoretical connotation of BRE as an “integrated regulatory capability”, and has better comparability with previous studies, the main results and discussion of this study are based on the total score of BRE.

## 4 Discussion

### 4.1 The relationship between coach emotional support and train disligently

The present study found that CES exerts a significant positive predictive effect on TD among college student-athletes; specifically, higher levels of perceived CES are associated with greater engagement, persistence, and willingness to exert effort in training among this population. Self-Determination Theory (SDT) posits that the satisfaction of an individual’s three basic psychological needs—autonomy, competence, and relatedness—fosters intrinsic motivation, thereby promoting sustained positive goal-directed behaviors (Ryan and Deci, 2000). In the present study, CES encompasses emotional care, belief encouragement, and empathetic understanding. High-quality CES can provide a secure psychological environment and core psychological resources for college student-athletes facing dual academic-training demands. In an environment characterized by respect and support, college student-athletes can develop positive cognitions that their training engagement is valued and their ability improvement is anticipated; they can also make bold attempts and achieve continuous breakthroughs in this non-judgmental setting. This psychological safety and positive cognitive link can effectively mitigate the negative impacts of academic-training conflict and strengthen the motivational chain of TD → ability improvement → self-actualization. Furthermore, CES can satisfy the three basic psychological needs of college student-athletes, namely autonomy, competence, and relatedness. When these three basic psychological needs are satisfied, individuals tend to pursue self-improvement and goal attainment, enhance the intensity of intrinsic motivation and frustration tolerance during training, and reduce negative behaviors such as passive participation and perfunctory engagement in training. Particularly for college student-athletes, who lack the constraints of professional contracts and rely more on intrinsic motivation to sustain their training behaviors, the intrinsic motivation potentially associated with CES is closely linked to the maintenance of long-term, high-intensity training engagement (Vealey, 1986). Thus, the present study reaffirms the positive effect of CES on TD among college student-athletes, which is consistent with the findings of previous studies (Bissett and Tamminen, 2022; Zhang and Khan, 2025). This further validates the stable positive predictive role of CES in training behaviors among athlete populations.

### 4.2 The mediating role of sport confidence

The present study found that SC plays a partial mediating role in the relationship between CES and TD among college student-athletes. This indicates that the effect of CES on TD among college student-athletes is not solely exerted through a direct pathway but is also indirectly transmitted via enhancing athletes’ SC—a key psychological bridge. First, CES can exert a significant positive effect on SC among college student-athletes. Emotional care, belief encouragement, and empathetic understanding inherent in CES can satisfy an individual’s three basic psychological needs—competence, relatedness, and autonomy (Ryan and Deci, 2000)—thereby enhancing their positive perceptions of their own athletic abilities, motivating them to actively accumulate successful experiences during training, and fostering the development of stable SC. Setbacks and performance fluctuations during training are prone to triggering self-doubt among athletes, whereas CES is essentially a positive feedback resource. By affirming the effort process, accepting attempts involving mistakes, mitigating athletes’ fear of failure, and strengthening the growth mindset that athletic abilities can be improved through training (Kegelaers and Wylleman, 2019; Huber, n.d.), it ultimately drives the enhancement of SC. This finding aligns with conclusions from previous studies that supportive environments constitute a core prerequisite for the cultivation of psychological capital (Felton and Jowett, 2013; Jowett et al., 2017). Second, SC can significantly and positively predict TD behaviors among college student-athletes. Studies have demonstrated that as a core psychological capital, SC generates positive self-efficacy, which drives individuals to exhibit greater initiative and persistence in goal pursuit (Feltz, 1988). Individuals with high SC possess strong ability beliefs and goal attainment expectations; they are able to frame training challenges as opportunities for ability enhancement rather than threats, thereby sustaining their engagement in TD. Meanwhile, according to Self-Efficacy Theory (Bandura and Wessels, 1997), individuals’ positive beliefs about their own abilities directly influence motivational intensity, effort levels, and frustration tolerance. College student-athletes with high SC are more inclined to set challenging training goals and are willing to exert extra effort to achieve these goals—a logical framework that has been fully validated in the present study.

### 4.3 The regulatory role of behavioral regulation in exercise

The present study further explored the moderating effect of BRE, and the results showed that this construct exerts a significant dual moderating effect on both the first stage (X → M) and the second stage (M → Y) of the mediating pathway. Moreover, as the level of BRE increases, the conditional indirect effect of the “X → M → Y” pathway gradually strengthens, with a moderated mediation index of 0.04 (95% CI = [0.020, 0.067]), which supports Hypotheses 3 and 4. It is important to note that before the moderation effect analysis, mean centering was performed on the continuous variables in this study. Therefore, the negative standardized *β* coefficients observed in the SC prediction model (CES: *β* = −0.895; BRE: *β* = −0.492) reflect only the direction of the influence of the deviation from the mean on the dependent variable, rather than the inherent directional relationship between the variables. Based on the zero-order correlation coefficients (CES and SC: r = 0.469**, BRE and SC: r = 0.403**), the unstandardized coefficients of the mediation paths (CES → SC: b = 0.512***), and the increasing trend of the conditional indirect effects (Table 6), it is clear that both CES and BRE have a significant positive predictive effect on SC, with the positive moderating effect of BRE further strengthening this relationship, which is entirely consistent with the research hypothesis. BRE is essentially an individual’s capability for active planning and dynamic adjustment of training-related cognition, emotions, and behaviors (Liu et al., 2025; Machida et al., 2012). For college student-athletes facing dual-task conflict between academics and training, the efficiency of their perception and utilization of external support resources is highly dependent on their own regulatory capabilities. College student-athletes with high levels of BRE are more adept at capturing and interpreting positive signals in CES through strategies such as goal decomposition and emotional regulation (Wilson et al., 2012; Hagger and Chatzisarantis, 2008). This efficient ability to convert support resources can enhance the satisfaction effect of CES on competence and relatedness, thereby accelerating the development of SC. Conversely, athletes with low levels of BRE are vulnerable to interference from dual pressures, making it difficult to establish an effective connection between CES and their own ability improvement. They may even overlook the positive value of support signals, resulting in inadequate conversion of support resources into psychological capital and ultimately weakening the promotional effect of CES on SC (Lox et al., 2019). SC serves as the psychological motivation driving training engagement; however, high confidence without behavioral regulation may lead to blind investment, thereby reducing training sustainability (Kegelaers and Wylleman, 2019). College student-athletes with high levels of BRE can balance confidence and training behaviors through rational regulation: they not only rely on high confidence to maintain training motivation but also achieve targeted rather than excessive engagement by dynamically monitoring training effects and flexibly adjusting strategies (Bahari et al., 2016; Ersöz and Eklund, 2017). At this point, the negative moderating effect of BRE is essentially a calibration effect—it prevents irrational training behaviors triggered by high confidence, ensures the effectiveness and sustainability of training engagement, and ultimately enhances overall training outcomes. In contrast, the training behaviors of athletes with low levels of BRE are more directly driven by confidence, lacking strategic adjustment. They may fall into reckless training when confidence is high and easily withdraw when confidence is low, which instead leads to volatility and inefficiency in training engagement. This finding expands the understanding of the role of BRE in previous studies. For practice, this means that BRE training should focus on both enhancing support utilization and calibrating confidence conversion, which has been reflected in the modular training courses proposed in the practical implications.

### 4.4 Limitations and prospects

First, the present study employed a cross-sectional research design, analyzing the relationships among variables through a one-time data collection process. This design limits the ability to interpret causal relationships between variables. Future studies could adopt a longitudinal research design to track individual changes over time, thereby facilitating the exploration of causal relationships among these variables. Additionally, a quasi-experimental design could be introduced to compare changes in athletes’ emotional control and skill development before and after intervention, thereby isolating the independent effect of emotional support training and strengthening causal inference. The analysis model should control for additional confounding variables that change over time, in order to reduce the influence of third variables on the observed relationships. Second, all variables in this study were measured using self-report scales. Although these scales are widely used in psychological research and possess sound reliability and validity, they rely solely on self-reported data, which may introduce self-report bias. Future research could consider integrating multiple data sources, such as observational assessments, peer evaluations, and objective measurement tools, to obtain more comprehensive and robust data. Finally, this study focused exclusively on the mediating role of SC and the moderating role of BRE, without incorporating other potential key variables. There were also limitations in terms of sample representativeness and gender balance. This limitation constrained the further improvement of the model’s explanatory power. Although certain variables were controlled for in the analysis, unaccounted confounding factors may still influence the study’s conclusions. Future studies should adopt targeted measures to expand the sample range, including university athletes from different regions and types of institutions, in order to enhance sample representativeness and the external validity of the results. Efforts should be made to balance the gender ratio in the sample and explicitly test the moderating effect of gender in the model, exploring potential differences between male and female athletes. Further investigation into other mediating or moderating factors is needed to explore underlying mechanisms, thereby enriching the empirical foundation of this research field. Additionally, other demographic variables should be incorporated into the analytical framework to comprehensively examine the model’s heterogeneous effects across different subgroups.

## 5 Conclusion and recommendations

This study, using Chinese college student-athletes as research subjects, constructed a moderated mediation model to explore the influence mechanism of Coach Emotional Support (CES) on Train Diligently (TD). It should be emphasized that all research conclusions are drawn from samples based on the Chinese cultural context and college student-athlete groups, and their generalization to other cultural contexts or athlete populations should be approached with caution. The unique cultural values, educational systems, and coach-athlete relationship patterns in China may influence the specific operation of the model, constituting an important boundary condition for the research results. Using methods including descriptive statistics, correlation analysis, regression analysis, and Bootstrap resampling tests, the following core conclusions were drawn: First, CES exerts a significant positive direct effect on TD among college student-athletes. Second, SC plays a partial mediating role between CES and TD among college student-athletes. Third, BRE exerts a dual significant moderating effect: it positively moderates the CES → SC pathway and negatively moderates the SC → TD pathway. Fourth, the overall moderated mediation model is valid.

Based on the research findings, the following recommendations are proposed for coaches, university sports management departments, and college student-athletes: At the coach level, attention should be given to implementing multidimensional emotional support and contextual communication strategies. For athletes facing dual pressures of academics and training, coaches should proactively engage in communication, patiently listening to their time constraints and psychological anxiety, and demonstrating empathy and understanding. For athletes underperforming in competitions, an emotion-first communication approach should be adopted, beginning with affirmation of their training efforts, followed by a joint analysis of the issues, avoiding direct criticism or questioning that may induce self-doubt in the athletes. Meanwhile, in daily training and communication, affirmative language should be used more frequently to help athletes build confidence and create a supportive training atmosphere. At the university sports management level, efforts should be made to establish a systematic behavioral regulation training system. This could be achieved through courses or specialized training sessions, guiding athletes to break down long-term training goals into actionable monthly, weekly, or even daily micro-goals, and providing goal-setting templates tailored to the characteristics of different sports. Simultaneously, athletes should be taught specific methods for strategy adjustment and emotional management during training. On this basis, a dual guidance mechanism involving coaches and peers should be established, integrating behavioral regulation guidance organically into daily training to ensure that theoretical guidance is effectively applied. At the college student-athlete level, efforts should be made to enhance psychological capital and strengthen behavioral regulation capabilities. It is recommended to record 1–2 psychological diaries per week, reviewing specific situations where the coach provided support, strengthening the psychological connection between coach support and self-worth, and accumulating positive experiences for self-regulation. When psychological needs are unmet, athletes should proactively and appropriately communicate with coaches, clearly expressing their status to facilitate more targeted support from the coach. Meanwhile, through a daily training adjustment log, athletes should record training goals, completion status, and encountered difficulties, dynamically optimizing subsequent training arrangements based on these records. When self-control is strong, athletes should be particularly adept at utilizing behavioral regulation strategies, avoiding blind training, monitoring their physical and mental state in real-time, and ensuring the sustainability and health of the training plan.

## Data Availability

The raw data supporting the conclusions of this article will be made available by the authors, without undue reservation.

## References

[ref1] Bahari F. Biyabani M. Zandi H. G. (2016). Relationship between mental toughness and behavioral regulation among university student-athletes. IOSR J. Sports Phys. Educ. 3, 06–10. doi: 10.9790/6737-03040610

[ref2] Bandura A. Wessels S. (1997). Self-Efficacy: The Exercise of Control. Freeman. Cambridge: Cambridge University Press.

[ref3] Barlow A. Banks A. P. (2014). Using emotional intelligence in coaching high-performance athletes: a randomised controlled trial. Coaching 7, 132–139. doi: 10.1080/17521882.2014.939679

[ref4] Bissett J. E. Tamminen K. A. (2022). Student-athlete disclosures of psychological distress: exploring the experiences of university coaches and athletes. J. Appl. Sport Psychol. 34, 363–383. doi: 10.1080/10413200.2020.1753263

[ref5] Carpentier J. Mageau G. A. (2016). Predicting sport experience during training: the role of change-oriented feedback in athletes’ motivation, self-confidence and needs satisfaction fluctuations. J. Sport Exerc. Psychol. 38, 45–58. doi: 10.1123/jsep.2015-0210, 27018557

[ref6] Contreira A. R. Nascimento Junior J. R. A. Caruzzo N. M. Nascimento Junior J. R. A. d. Costa L. C. A. Gaion P. A. . (2019). Basic psychological needs and sports satisfaction among Brazilian athletes and coaches: the mediating role of the dyadic relationship. Front. Psychol. 10:2543. doi: 10.3389/fpsyg.2019.02543, 31781009 PMC6861456

[ref7] De Cooman R. De Gieter S. Pepermans R. Jegers M. Van Acker F. (2009). Development and validation of the work effort scale. Eur. J. Psychol. Assess. 25, 266–273. doi: 10.1027/1015-5759.25.4.266

[ref8] Durand-Bush N. Salmela J. H. (2002). The development and maintenance of expert athletic performance: perceptions of world and Olympic champions. J. Appl. Sport Psychol. 14, 154–171. doi: 10.1080/10413200290103473

[ref9] Edmunds J. Ntoumanis N. Duda J. L. (2006). A test of self-determination theory in the exercise domain. J. Appl. Soc. Psychol. 36, 2240–2265. doi: 10.1111/j.0021-9029.2006.00102.x

[ref10] Ersöz G. Eklund R. C. (2017). Behavioral regulations and dispositional flow in exercise among American college students relative to stages of change and gender. J. Am. Coll. Heal. 65, 94–102. doi: 10.1080/07448481.2016.1239203, 27661351

[ref11] Felton L. Jowett S. (2013). Attachment and well-being: the mediating effects of psychological needs satisfaction within the coach–athlete and parent–athlete relational contexts. Psychol. Sport Exerc. 14, 57–65. doi: 10.1016/j.psychsport.2012.07.006

[ref12] Feltz D. L. (1988). Self-confidence and sports performance. Exerc. Sport Sci. Rev. 16, 423–458, 3292264

[ref13] Ferreira J. G. Rodrigues F. Sobreiro P. Silva M. Santos F. J. Carvalho G. . (2024). Social support, network, and relationships among coaches in different sports: a systematic review. Front. Psychol. 15:1301978. doi: 10.3389/fpsyg.2024.1301978, 39380751 PMC11458428

[ref14] Garrido-Muñoz M. Blanco-García C. Diez-Vega I. García-Merino S. Acebes-Sánchez J. . (2024). Psychological resilience, athletic experience, and competitive level of judokas. A transversal study. Front. Psychol. 15:1440412. doi: 10.3389/fpsyg.2024.1440412 39144597 PMC11321973

[ref15] Hagger M. Chatzisarantis N. (2008). Self-determination theory and the psychology of exercise. Int. Rev. Sport Exerc. Psychol. 1, 79–103. doi: 10.1080/17509840701827437

[ref16] Hays K. Thomas O. Maynard I. Bawden M. (2009). The role of confidence in world-class sport performance. J. Sports Sci. 27, 1185–1199. doi: 10.1080/02640410903089798, 19724964

[ref71] Hayes A. F. (2013). Mediation, moderation, and conditional process analysis[J]. Introduction to mediation, moderation, and conditional process analysis:. A regression-based approach, 1, 12–20.

[ref17] Huber J. J. (n.d.). Applying Educational Psychology in Coaching Athletes. Champaign, IL: Human Kinetics.

[ref18] Jowett S. Adie J. W. Bartholomew K. J. Yang S. X. Gustafsson H. Lopez-Jiménez A. . (2017). Motivational processes in the coach-athlete relationship: a multi-cultural self-determination approach. Psychol. Sport Exerc. 32, 143–152. doi: 10.1016/j.psychsport.2017.06.004

[ref19] Kegelaers J. Wylleman P. (2019). Exploring the coach’s role in fostering resilience in elite athletes. Sport Exerc. Perform. Psychol. 8:239. doi: 10.1037/spy0000151

[ref20] Lafrenière M. A. K. Jowett S. Vallerand R. J. Carbonneau N. (2011). Passion for coaching and the quality of the coach–athlete relationship: the mediating role of coaching behaviors. Psychol. Sport Exerc. 12, 144–152. doi: 10.1016/j.psychsport.2010.08.002

[ref21] Li X. Sun J. Han J. (2025). The relationship between coaches leadership behaviour and training attitudes of college high-level athletes: the role of self-management and sport self-efficacy. BMC Psychol. 13:1336. doi: 10.1186/s40359-025-03703-y, 41361322 PMC12683865

[ref22] Liu J. D. Chung P. K. Zhang C. Q. Si G. (2015). Chinese-translated behavioral regulation in exercise questionnaire-2: evidence from university students in the mainland and Hong Kong of China. J. Sport Health Sci. 4, 228–234. doi: 10.1016/j.jshs.2014.03.017

[ref23] Liu H. J. De Jonge K. M. M. Den Hartigh R. J. R. Yperen N. W. (2025). Basic psychological need support, need satisfaction, and autonomous motivation in coach-athlete relationships: a systematic review and meta-analysis. Int. J. Sports Sci. Coach. 2025:17479541251400621. doi: 10.1177/17479541251400621

[ref24] López-Walle J. Balaguer I. Castillo I. Tristán J. (2012). Autonomy support, basic psychological needs and well-being in Mexican athletes. Span. J. Psychol. 15, 1283–1292. doi: 10.5209/rev_sjop.2012.v15.n3.39414, 23156932

[ref25] Lorimer R. Jowett S. (2013). “Empathic understanding and accuracy in the coach–athlete relationship,” in Paul Potrac, Wade Gilbert, Jim Denison eds., Routledge Handbook of Sports Coaching, (London: Routledge), 321–332.

[ref26] Lox C. L. Ginis K. A. M. Gainforth H. L. (2019). The Psychology of Exercise: Integrating Theory and Practice. New York: Routledge.

[ref27] Machida M. Marie Ward R. Vealey R. S. (2012). Predictors of sources of self-confidence in collegiate athletes. Int. J. Sport Exerc. Psychol. 10, 172–185. doi: 10.1080/1612197x.2012.672013

[ref28] Machida-Kosuga M. Kohno N. (2023). Athlete leader development: the perspectives of athlete leaders, teammates, and coaches. J. Appl. Sport Psychol. 35, 111–135. doi: 10.1080/10413200.2021.2024624

[ref29] Mageau G. A. Vallerand R. J. (2003). The coach–athlete relationship: a motivational model. J. Sports Sci. 21, 883–904. doi: 10.1080/0264041031000140374 14626368

[ref30] Markland D. Tobin V. (2004). A modification to the behavioural regulation in exercise questionnaire to include an assessment of amotivation. J. Sport Exerc. Psychol. 26, 191–196. doi: 10.1123/jsep.26.2.191

[ref31] Rocchi M. Pelletier L. (2018). How does coaches’ reported interpersonal behavior align with athletes’ perceptions? Consequences for female athletes’ psychological needs in sport. Sport Exerc. Perform. Psychol. 7:141. doi: 10.1037/spy0000116

[ref32] Ryan R. M. Deci E. L. (2000). Self-determination theory and the facilitation of intrinsic motivation, social development, and well-being. Am. Psychol. 55, 68–78. doi: 10.1037/0003-066X.55.1.68, 11392867

[ref33] Ryan R. M. Deci E. L. (2024). “Self-determination theory,” in Encyclopedia of Quality of Life and Well-Being Research, ed. Michalos A. C. (Cham: Springer International Publishing), 6229–6235.

[ref34] Shen L. (2004). Empirical Examination of the Structural Model of Athletic Self-Confidence Among Chinese Athletes. Beijing: Beijing Sport University.

[ref35] Simons E. E. Bird M. D. (2023). Coach-athlete relationship, social support, and sport-related psychological well-being in National Collegiate Athletic Association Division I student-athletes. J. Study Sports Athletes Educ. 17, 191–210. doi: 10.1080/19357397.2022.2060703

[ref36] Tang D. Wen Z. (2020). Common method bias testing: issues and recommendations. Psychol. Sci. 43, 215–223. Available at: https://www.who.int/news-room/fact-sheets/detail/anaemia.

[ref37] Teixeira P. J. Carraça E. V. Markland D. Silva M. N. Ryan R. M. (2012). Exercise, physical activity, and self-determination theory: a systematic review. Int. J. Behav. Nutr. Phys. Act. 9:78. doi: 10.1186/1479-5868-9-78, 22726453 PMC3441783

[ref38] Trigueros R. Aguilar-Parra J. M. Álvarez J. F. González-Bernal J. J. López-Liria R. (2019). Emotion, psychological well-being and their influence on resilience. A study with semi-professional athletes. Int. J. Environ. Res. Public Health 16:4192. doi: 10.3390/ijerph16214192, 31671532 PMC6861950

[ref39] Vealey R. S. (1986). Conceptualization of sport-confidence and competitive orientation: preliminary investigation and instrument development. J. Sport Psychol. 8, 221–246. doi: 10.1123/jsp.8.3.221

[ref40] Wei X. Xu Q. (2022). Predictors of willingness to communicate in a second language (L2 WTC): toward an integrated L2 WTC model from the socio-psychological perspective. Foreign Lang. Ann. 55, 258–282. doi: 10.1111/flan.12595

[ref41] Wilson P. M. Sabiston C. M. Mack D. E. Blanchard C. M. (2012). On the nature and function of scoring protocols used in exercise motivation research: an empirical study of the behavioral regulation in exercise questionnaire. Psychol. Sport Exerc. 13, 614–622. doi: 10.1016/j.psychsport.2012.03.009

[ref42] Wylleman P. (2000). Interpersonal relationships in sport: uncharted territory in sport psychology research. Int. J. Sport Psychol. 31, 555–572.

[ref43] Zhang Y. Fan R. (2025). Effects of coaches’ authentic leadership on athletes’ training competition satisfaction: the mediating roles of psychological ownership and athlete engagement. Int. J. Sports Sci. Coach. 20, 79–91. doi: 10.1177/17479541241267853

[ref44] Zhang X. Khan S. U. (2025). Fostering self-efficacy in Chinese university athletes: the mediating roles of psychological resilience and emotional regulation and the moderating role of autonomy-supportive coaching. Front. Psychol. 16:1664339. doi: 10.3389/fpsyg.2025.1664339, 41473516 PMC12746662

